# Tratamento Percutâneo da Insuficiência Mitral Secundária por MitraClip: Mitra-FR *versus* COAPT

**DOI:** 10.36660/abc.20200063

**Published:** 2021-05-06

**Authors:** Sergio Barros-Gomes, Flávio Tarasoutchi, Ana Clara Tude Rodrigues, Lara Ferreira Nhola, Pedro Alves Lemos, Samira Saady Morhy, Claudio Henrique Fischer, Marcelo Luiz Campos Vieira

**Affiliations:** 2 Hospital Israelita Albert Einstein São PauloSP Brasil Hospital Israelita Albert Einstein, São Paulo, SP - Brasil; 1 Universidade de São Paulo Instituto do Coração São PauloSP Brasil Instituto do Coração da Universidade de São Paulo, São Paulo, SP - Brasil; 3 Universidade Federal de São Paulo São PauloSP Brasil Universidade Federal de São Paulo, São Paulo, SP - Brasil

**Keywords:** Insuficiência Cardiaca, Insuficiência da Válvula Mitral, Ecocardiografia/métodos, Ensaios Clínicos

## Introdução

A insuficiência mitral (IM) secundária, ou funcional, deve-se a alterações na geometria do ventrículo esquerdo (VE) secundária à disfunção ventricular.^1^ Ocorre quando uma doença isquêmica cardíaca ou uma cardiopatia dilatada de qualquer etiologia causa dilatação do VE, dilatação do anel mitral e/ou deslocamento do músculo papilar, resultando em má coaptação das cúspides valvares e refluxo valvar.^2^ Dados estatísticos da American Heart Association indicam que 16.250 por milhão de americanos tenham IM secundária,^3^^,^^4^ números que totalizam mais de 5 milhões de casos somente nos EUA, e estima-se que esse número seja ainda maior tendo em vista o contínuo crescimento e envelhecimento da população. Isso é importante, uma vez que a IM secundária acarreta um mau prognóstico e é preditor independente de mortalidade.^5^^,^^6^

Por muitos anos, a intervenção mecânica da IM secundária (cirúrgica ou percutânea) foi restrita a casos refratários ao tratamento clínico convencional,^7^^,^^8^ com evidência respaldada principalmente por dois importantes estudos do grupo Cardiothoracic Surgical Trials Network.^9^^,^^10^ O primeiro estudo^9^ randomizou 301 pacientes com IM isquêmica de grau moderado e não encontrou diferenças na geometria ventricular entre os pacientes que receberam revascularização cirúrgica do miocárdico *versus* a combinação revascularização cirúrgica e reparo valvar mitral. O segundo estudo^10^ estudou 251 pacientes com IM grave e não encontrou diferenças com relação à mortalidade, além da maior recorrência de insuficiência mitral e taxas de complicações entre os pacientes tratados com plastia da válvula mitral *versus* troca valvar. Por causa desses dois estudos, as recomendações da American Heart Association/American College of Cardiology^7^ e a Diretriz Brasileira de Valvopatias^8^ classificaram a intervenção cirúrgica ou percutânea da válvula mitral como Classe IIb de indicação.

Até recentemente, nenhum estudo randomizado havia comparado a intervenção percutânea da IM secundária com o tratamento clínico convencional. Em 2018, a conduta frente à IM secundária mudou decisivamente com a apresentação de dois ensaios clínicos randomizados: o Multicentre Study of Percutaneous Mitral Valve Repair MitraClip Device in Patients with Severe Secondary Mitral Regurgitation (MITRA-FR)^11^ e o Cardiovascular Outcomes Assessment of the MitraClip Percutaneous Therapy for Heart Failure Patients with Functional Mitral Regurgitation (COAPT).^12^ Esses estudos avaliaram a eficácia e a segurança de duas estratégias terapêuticas em pacientes com IM secundária importante – a terapia percutânea com MitraClip® em conjunto com tratamento clínico otimizado *versus* tratamento clínico otimizado isolado.

Neste artigo, abordaremos as principais similaridades e diferenças entre os dois estudos e faremos considerações a respeito da aplicação desse procedimento na prática clínica, incluindo o perfil ideal do candidato para o procedimento (Tabela 1).

**Tabela 1:** Características de recrutamento, randomização e seguimento clínico

Variável		MITRA-FR	COAPT
Pacientes, n		304	614
Pacientes intervenção/Controle, n		152/152	302/312
Tempo de estudo, anos		2013-2017	2012-2017
**Critério de inclusão**			
	- ERO, mm^2^	>20	>30
	- VR, ml/batimento	30	45
	- FEVE, %	15-40	20-50
	- DSFVE, mm	NA	≤70
	- Medicações de base	Ajustadas em cada braço de acordo com a prática clínica	Dose máxima estabilizada e terapia de ressincronização se apropriado
	- Sintomas	NYHA II, III, IV	NYHA II, III, IV

DSFVE: diâmetro sistólico final do ventrículo esquerdo; ERO: orifício regurgitante efetivo; FEVE: fração de ejeção do ventrículo esquerdo; NYHA: New York Heart Association; VR: volume regurgitante.

### MITRA-FR

O MITRA-FR foi um estudo multicêntrico em 37 centros franceses que randomizou 304 pacientes com IM secundária importante, insuficiência cardíaca (IC) sistólica sintomática e fração de ejeção do ventrículo esquerdo (FEVE) entre 15% e 40% em duas estratégias terapêuticas, na razão de 1:1, alocados para tratamento percutâneo com MitraClip® em conjunto com tratamento clínico otimizado (grupo intervenção; 152 pacientes) *versus* tratamento clínico otimizado isolado (grupo controle; 152 pacientes).^11^ A IM secundária importante foi definida como tendo área do orifício regurgitante efetivo (ERO) >20 mm^2^ ou volume regurgitante (VR) >30 mL por batimento. O desfecho primário foi mortalidade por qualquer causa ou hospitalização por IC dentro de 12 meses. Os pacientes dos dois grupos apresentaram uma melhora da classe funcional, mas sem diferença significativa entre os dois grupos. Por fim, não houve diferença significativa no desfecho primário combinado (54,6% *vs.* 51,3%, respectivamente; p = 0,53), taxa de mortalidade (24,3% *vs.* 22,4%; p >0,05) e taxa de hospitalização (48,7% *vs.* 47,4%; p >0,05) entre o grupo intervenção *versus* controle durante 1 ano de seguimento.**** Da mesma forma, não houve diferença significativa no desfecho primário combinado (63,8% *vs.* 67,1%, respectivamente; p >0,05), taxa de mortalidade (34,9% *vs.* 34,2%; p >0,05) e taxa de hospitalização (55,9% *vs.* 61,8%; p >0,05) entre o grupo intervenção *versus* controle durante 2 anos de seguimento.^13^ Os autores concluíram que o MitraClip® é seguro e efetivo na IM secundária em comparação ao tratamento clínico otimizado, mas sem melhora na sobrevida ou redução da hospitalização por IC em pacientes com IM secundária e IC sistólica.

### COAPT

O COAPT foi um estudo multicêntrico que randomizou 614 pacientes em 78 centros americanos e canadenses com IC sistólica sintomática e IM secundária moderada a importante (3+) ou importante (4+), definida como ERO >30 mm^2^ ou VR >45 mL por batimento, com FEVE ≥20% (FEVE média 31,3 ± 9,3%), na razão de 1:1, alocados para tratamento percutâneo com MitraClip® em conjunto com tratamento clínico otimizado (grupo intervenção; 302 pacientes) *versus* tratamento clínico otimizado isolado (grupo controle; 312 pacientes).^12^ IC sintomática foi definida como sintomas de IC apesar da dose máxima medicamentosa tolerada. O desfecho primário de eficácia foi hospitalização por IC dentro de 24 meses, e o desfecho primário de segurança foi evento livre de complicações relacionadas ao MitraClip® em 12 meses. A taxa anual de hospitalização por IC dentro de 24 meses foi 35,8% por paciente/ano no grupo intervenção *versus* 67,9% no grupo controle (*hazard ratio* 0,53; 95% IC 0,40-0,70; p <0,001). A porcentagem de evento-livre de complicações relacionadas ao dispositivo em 12 meses foi 96,6% (p <0,001), enquanto morte por qualquer causa em 24 meses ocorreu em 29,1% no grupo intervenção em comparação a 46,1% no grupo controle (*hazard ratio* 0,62; 95% IC, 0,46-0,82; p <0,001). O grupo intervenção não somente reduziu a taxa de hospitalizações por IC em 47%, mas também reduziu a mortalidade em 38%. A redução no risco absoluto da mortalidade por todas as causas no grupo MitraClip® foi 17%, e o número necessário para prevenir uma morte em 2 anos foi 5,9; já para prevenir uma hospitalização por IC em 2 anos foi 3,1. Os autores concluíram que a terapia combinada MitraClip® e tratamento clínico otimizado em pacientes com IC sistólica sintomática e IM moderada a importante ou importante reduz o número de hospitalizações por IC e a mortalidade por todas as causas em 2 anos, quando comparada com tratamento clínico otimizado exclusivamente. As Tabelas 2 e 3 comparam as características e os desfechos clínicos entre os dois estudos.

**Tabela 2:** Características clínicas e ecocardiográficas

Variável^a^		MITRA-FR	COAPT
**Clínica**			
Idade, anos			
	- Braço MitraClip®	70 ± 10	72 ± 12
	- Braço controle	71 ± 10	73 ± 10
Sexo, masculino, n (%)		120 (79)	201 (67)
		107 (70)	(62)
NYHA, %			
	- I	0	0,2
	- II	32,9	39
	- III	58,5	52,5
	- IV	8,6	8,3
Cardiomiopatia isquêmica			
	- Braço MitraClip®	95 (62,5)	184 (60,9)
	- Braço controle	85 (56,3)	189 (60,6)
Revascularização coronariana prévia			
	- Braço MitraClip®	71 (46,7)	PCI: 130 (43,0) CABG: 121 (40,1)
	- Braço controle	62 (42,4)	PCI: 153 (49,0) CABG: 126 (40,4)
Ressincronização cardíaca prévia			
	- Braço MitraClip®	46 (30,5)	115 (38,1)
	- Braço controle	35 (23,0)	109 (34,9)
Risco Cirúrgico			
	- Escore STS	NA	8,2 ± 5,9%
	- EuroScore II	6,2 (3,5-11)	NA
**Ecocardiográfica**			
Gravidade da IM, %			
	- ERO 20-29 mm^2^ (moderada)	157 (52,2)	80 (13,5)
	- ERO 30-39 mm^2^ (moderada/importante)	95 (31,6)	270 (45,7)
	- ERO ≥ 40 mm^2^ (importante)	49 (16,3)	241 (40,8)
ERO, mm^2^		31 ± 10	41 ± 15
VDFVEI, mL/m^2^		135 ± 35	101 ± 34
FEVE, %		33 ± 7	31 ± 9

ERO: orifício regurgitante efetivo; FEVE: fração de ejeção do ventrículo esquerdo; NA: não aplicável; NYHA: New York Heart Association; STS: Society Toracic of Surgery – risco de morte em 30 dias após troca valvar mitral; VDFVEI: volume diastólico final do ventrículo esquerdo indexado.

a Variáveis categóricas são reportadas em números (porcentagens); variáveis contínuas são reportadas como média ± SD e mediana (variação interquartil).

**Tabela 3:** Desfecho clínico

Variável^a^		MITRA-FR	COAPT
Grupo MitraClip® apenas, n			
	- Complicações no procedimento	21 (14,6)	25 (8,5)
	- IM ≥ +2 na alta	93 (24,4)	214 (17,7)
	- IM ≥ +2 em 2 anos	48 (49,5)^b^	26 (22,8)
Mortalidade por qualquer causa em 2 anos, n			
	- Braço MitraClip®	53 (34,9)	80 (29,1)
	- Braço controle	52 (34,2)	121 (46,1)
	Valor p	>0,05	<0,001
Hospitalizações por ICC em 2 anos, n			
	- Braço MitraClip®	85 (55,9)	92 (35,7)
	- Braço controle	94 (61,8)	151 (56,7)
	Valor p	>0,05	<0,001
Mortalidade por qualquer causa ou hospitalizações associadas a IC em 2 anos, n			
	- Braço MitraClip®	97 (63,8)	129 (45,7)
	- Braço controle	102 (67,1)	191 (67,9)
	Valor p	>0,05	<0,001

IC: insuficiência cardíaca; ICC: insuficiência cardíaca congestiva; IM: insuficiência mitral.

a Variáveis categóricas são reportadas em números (porcentagens).

b IM ≥ +2 em 1 ano.

### Principais Similaridades e Diferenças

Os dois ensaios tiveram resultados conflitantes, com o COAPT mostrando benefício do MitraClip® *versus* terapia medicamentosa, enquanto o MITRA-FR não mostrou benefício relacionado ao MitraClip®. Não há dúvida de que esses dois estudos mudaram nosso entendimento de IM secundária. No entanto, por que eles apresentaram resultados significativamente diferentes? Por que o estudo COAPT teve um resultado positivo, enquanto o MITRA-FR foi neutro? Provavelmente as repostas a tais questões são multifatoriais e incluem diferenças na seleção de pacientes, otimização da terapia medicamentosa, grau da IM e no remodelamento ventricular.

#### Recrutamento:

O recrutamento do COAPT foi mais seletivo em comparação ao MITRA-FR, visto que seu recrutamento foi mais lento e prolongado. O número de pacientes foi diferente nos dois estudos: COAPT recrutou cerca de 300 pacientes em cada braço, e o MITRA-FR cerca de 150 pacientes. Talvez o tamanho amostral da população do MITRA-FR, após exclusão de pacientes com seguimento incompleto, possa não ter sido suficiente para detectar significância estatística e, dessa forma, evitar o erro estatístico tipo II, principalmente em relação aos desfechos secundários. No estudo COAPT, o número de hospitalizações entre as duas estratégias terapêuticas divergiu desde o início do seguimento, parcialmente explicado pelo tratamento medicamentoso mais rigoroso.

#### Grau da IM:

No MITRA-FR, a média de ERO foi 31 mm^2^, enquanto o COAPT teve média de ERO de 41 mm.^2^ Apesar de o critério de inclusão de ambos os estudos ter sido IM ao menos moderada a importante, o estudo COAPT seguiu as recomendações americanas de 2008,^14^ que classifica como IM moderada a importante quando o ERO é ≥ 30 mm^2^ e/ou VR de 45 mL; já o MITRA-FR seguiu as recomendações europeias de 2012:^15^ ERO ≥ 20 mm^2^ e/ou VR de 30 mL como IM moderada a importante. Essa discordância é baseada no conceito de que a mortalidade em pacientes com IM secundária é significativamente maior para níveis menores de ERO e VR.^16^^,^^17^ Entretanto, o mecanismo da IM funcional é complexo e não se sabe se ERO ou VR moderados atuam ativamente como causadores do remodelamento e disfunção ventricular, ou se são meros marcadores resultantes da cardiomiopatia incipiente. As diretrizes subsequentes retornaram o ERO e o VR ao valor habitual; com base nas recomendações atuais, ERO de 30 mm^2^ é considerado moderado, enquanto ERO ≥ 40 mm^2^ é importante.^8^^,^^18^ Novos estudos sugerem que a abordagem unificada – baseada na integração de ERO, VR e fração regurgitante (FR) – possa ser um excelente discriminador de IM secundária importante quando comparada aos algoritmos estabelecidos nas últimas diretrizes e, portanto, um excelente identificador de pacientes com alto risco.^19^ Dito isso, um número significativo de pacientes (52%) com IM moderada (ERO 20 a 30 mm^2^) foi recrutado no MITRA-FR, enquanto apenas 14% dos pacientes com essas características foram recrutados no COAPT. Já em relação à IM importante (ERO ≥ 40 mm^2^), apenas 16% dos pacientes do MITRA-FR tinham IM importante *versus* 41% do COAPT. Os achados dos dois estudos sugerem que o benefício do MitraClip® é maior nos pacientes com ERO > 40 mm^2^ (*i. e.*, IM verdadeiramente importante).

#### Remodelamento Ventricular:

O volume diastólico final do ventrículo esquerdo indexado (VDFVEI) médio dos pacientes do estudo MITRA-FR foi 135 mL/m^2^ comparado a 101 mL/m^2^ do COAPT. O VE foi significativamente maior no MITRA-FR, caracterizando ventrículos mais remodelados, em estágios mais avançados de cardiomiopatia. Essa diferença é provavelmente devido à exclusão de pacientes com dilatação/disfunção severa no COAPT (diâmetro sistólico do VE >70 mm), enquanto no MITRA-FR não havia essa limitação. O critério de inclusão da FEVE entre os dois estudos também foi diferente: o COAPT incluiu pacientes com FEVE 20% a 50% *versus* FEVE de 10% a 40% no MITRA-FR. Interessantemente, um subgrupo de pacientes do estudo COAPT que não se beneficiou do tratamento por MitraClip® (número de hospitalizações associadas a IC em 12 meses) foi o dos pacientes com ERO e VDFVEI relativamente semelhantes àqueles recrutados no estudo MITRA-FR (ERO ≥ 30 mm^2^ e VDFVEI >96 mL/m^2^).^20^ Esses fatos sugerem que pacientes com IM moderada, VE marcadamente mais dilatados e com maior disfunção possam não ser os candidatos ideais para o tratamento com MitraClip®. De fato, a alta recorrência de IM e o pior desfecho clínico já haviam sido reportados previamente na correção cirúrgica de pacientes com IM isquêmica, dilatação ventricular (diâmetro diastólico do VE >65 mm) e disfunção grave do VE (FEVE <20% e diâmetro sistólico do VE >55 mm).^21^^,^^22^ No estudo MITRA-FR, a cardiomiopatia foi, possivelmente, o principal causador dos sintomas de IC e, consequentemente, o determinante do desfecho clínico desfavorável, ou seja, a IM foi meramente um fator secundário ao remodelamento ventricular. Em contrapartida, no COAPT, a IC foi, em parte, decorrente da IM e, por isso, o grau da IM no estudo COAPT foi maior, enquanto a cardiomiopatia foi menos avançada (VE de dimensão menor e FEVE maior).

#### Terapia Medicamentosa e Otimização Terapêutica:

No estudo COAPT, o critério de inclusão de pacientes foi IC sistólica sintomática apesar da dose máxima medicamentosa tolerada, uso de terapia de ressincronização, uso de desfibriladores e terapia de revascularização cardíaca (se apropriado). Os pacientes foram otimizados clinicamente antes do recrutamento, e apenas poucos ajustes de medicação foram feitos no decorrer do seguimento. Já no MITRA-FR, não foi possível otimizar a medicação em todos os pacientes antes da randomização, e múltiplos reajustes ocorreram ao longo do seguimento. No MITRA-FR, a medicação foi ajustada pelos investigadores, enquanto no COAPT a medicação foi ajustada mais rigorosamente por um grupo de especialistas que supervisionavam a dose máxima tolerada, antes e após a intervenção. A posologia inicial e as doses ajustadas de cada medicação foram contabilizadas no estudo COAPT. Este rigor em termos de posologia e otimização medicamentosa implementada no estudo COAPT certamente não reflete a prática clínica diária.

#### Sucesso na Redução da IM:

No final de 12 meses, 83% dos pacientes do MITRA-FR tiveram IM ≤ +2 (moderada) em comparação a 95% dos pacientes do COAPT. Consequentemente, 17% dos pacientes do MITRA-FR tiveram IM ≥ +3 (moderada/importante) em 12 meses em comparação a 5% dos pacientes do COAPT. O estudo COAPT teve uma estratégia mais agressiva em termos de clipes implantados quando comparado ao MITRA-FR (uso de um clipe em 36% dos casos para o COAPT *vs.* 46% para o MITRA-FR; dois clipes em 55% dos casos do COAPT *vs.* 45% para o MITRA-FR; três clipes em 8% dos pacientes do COAPT *vs.* 9% do MITRA-FR; quatro clipes em 0,3% dos pacientes do COAPT *vs.* 0% para o MITRA-FR). É possível que a maior taxa de sucesso na redução da IM possa estar associada a resultados favoráveis.

#### Fisiopatologia:

Divergências em termos de fisiopatologia foram elegantemente demonstradas por Packer e Grayburn et al.,^23^ que apresentaram o conceito de IM proporcional *versus* IM desproporcional com base na combinação de ERO e volume diastólico final (VDF) – razão ERO/VDF. Assumindo uma FEVE de 30% e uma fração regurgitante de 50% (perfil dos pacientes dos estudos), os autores mostraram graficamente que um ERO de 30 mm^2^ e um VDF maior (entre 220 e 240 mL) poderia resultar em uma fração regurgitante de 50%, assim como um ERO de 20 mm^2^ e um VDF normal poderiam resultar em uma fração regurgitante de 50%.^23^ Os autores sugerem que o tratamento percutâneo da válvula mitral pelo MitraClip® seja mais benéfico em pacientes com IM desproporcional ao tamanho do VE – isto é, quando a IM for maior do que a esperada para um VE dilatado, o tratamento com MitraClip® poderá ter um resultado mais favorável (ERO maior e VE menor). Em contrapartida, IM proporcional representaria pacientes mais doentes, com ventrículos maiores e menor grau de IM – em outras palavras, pacientes com cardiomiopatia em estágio avançado tardiamente selecionados para o tratamento intervencionista.

No entanto, Gaasch e Meyer et al.,^24^ sugeriram que a gravidade da IM entre os dois estudos não seja tão diferente e que, de fato, seja semelhante. Os autores advogam que a fisiopatologia da IM é melhor descrita através do VR (ou a fração regurgitante) em vez do ERO. O VR é determinado pelo ERO e pela magnitude e duração do gradiente de pressão sistólica através da válvula regurgitante, ou seja, o ERO é apenas uma das variáveis determinantes do VR. É o VR que afeta o tamanho do VE a uma determinada FEVE, e exibe uma relação direta com o VDF. Desse modo, eles propuseram graficamente que a associação entre gravidade da IM e tamanho do VE seja baseada no conceito entre VR e VDF – razão VR/VDF –; o seu quociente é corrigido de maneira uniforme, tornando-o um índice adimensional. Assumindo uma fração regurgitante de 50% no estudo COAPT (suposição baseada na FEVE e em dados ecocardiográficos) e fração regurgitante de 53% fornecida no MITRA-FR, a razão VR sobre VDF (VR/VDF) foi de 0,18 e 0,15, respectivamente. Esses coeficientes de proporcionalidade são relativamente baixos (ambos <0,20) e semelhantes aos valores reportados em estudos prévios de IM secundária, refletindo uma contribuição proporcionalmente pequena do VR para um VDF grande. Assim, há um aumento desproporcional do VE no perfil dos pacientes dos dois estudos tipicamente visto em pacientes com IM secundária (IM desproporcional) em comparação aos pacientes com IM primária (VDF proporcional ao VR).

#### Volumes subestimados:

No estudo COAPT, os pacientes tiveram um ERO médio de 41 ± 15 mm^2^, o que corresponde a um VR de no mínimo 45 a 60 mL. O volume ejetado total do VE no estudo COAPT foi 57 mL (volume diastólico final do VE subtraído pelo volume sistólico final), o que é totalmente incompatível para manter um débito cardíaco satisfatório. Assumindo um volume ejetado total do VE de 57 mL, então o VR é o volume ejetado total do VE subtraído pelo volume ejetado para a via de saída (ou seja, o volume ejetado total do VE é igual ao VR mitral mais o volume ejetado na via de saída). Com base nesses cálculos, o volume ejetado para a frente é de 0 a 15 mL, o que seria incompatível com a vida. Está claro que o VDF no estudo COAPT está subestimado. Se assumirmos um ERO de 41 mm^2^ e um VR de 60 mL (aproximado do estudo COAPT), então o VDF deveria ser maior que 300 mL (assumindo uma fração regurgitante de 50% e FEVE de 31% como relatado no estudo). De qualquer forma, o diâmetro diastólico do VE foi menor no estudo COAPT (média de 69 mm no MITRA-FR *vs.* 62 mm no COAPT) confirmando VE menores.

De fato, a quantificação da IM secundária através do ecocardiograma bidimensional é desafiadora devido a inúmeras limitações do próprio método, além da complexa fisiopatologia da IM. Em pacientes com IM funcional, em sua grande maioria, o ERO e o VR pelo método PISA são subestimados com os valores da ressonância cardíaca^25^ e ecocardiografia tridimensional.^26^ O orifício não circular e o comportamento dinâmico da IM contribuem significativamente para essas diferenças. Talvez a fração regurgitante possa suprir essas limitações e corroborar como variável essencial de gravidade, além do seu importante papel prognóstico.^12^ A fração regurgitante é calculada pela razão do VR sobre o volume ejetado total (VR/volume ejetado total), que, apesar de serem variáveis dependentes de condições de carga, tamanho e função do VE, o seu quociente é corrigido de maneira uniforme por esses parâmetros, podendo assim ser um indicador mais robusto.^27^

#### *Outros fatores*:

Vale lembrar que, diferentemente da IM primária, em que sua gravidade é puramente quantificada com base no grau da IM, a IM secundária é complexa, heterogênea e influenciada por vários fatores: idade, doença de base subjacente, comorbidades, remodelamento do VE, extensão do infarto, distúrbios hemodinâmicos, entre outros.^28^ No estudo COAPT, o desfecho combinado mortalidade ou internação por insuficiência cardíaca no braço que recebeu terapia por MitraClip® foi relativamente alto (46%). Isso mostra que, independentemente do reparo da válvula, esses pacientes continuam tendo um prognóstico reservado, visto que boa parte do risco é relacionada a esses fatores.

Nessa mesma linha, Cavalcante et al.^29^ revelaram que a fração regurgitante e o tamanho do infarto medidos em pacientes com cardiopatia isquêmica é um importante estratificador de risco que vai além do tamanho do VE e outras variáveis clínicas. Os autores também mostraram que o prognóstico desses pacientes é pior à medida que o tamanho do infarto e do grau de IM aumentam. Interessantemente, a extensão da fibrose não foi medida nos estudos MITRA-FR e COAPT, mas acreditamos que certamente teve impacto clínico no desfecho desses estudos. Talvez, pacientes com um coração maior e com uma maior área de infarto não possam se beneficiar do MitraClip®; do mesmo modo, podemos especular que os pacientes do estudo MITRA-FR continham maior área de fibrose e, por isso, menor benefício com terapia por MitraClip®. Novos estudos correlacionando desfechos clínicos em pacientes tratados por MitraClip® e extensão da fibrose seriam interessantes.

### Implicações na Prática Clínica

Os dois estudos avaliaram a mesma entidade clínica: a IM funcional ou secundária. No COAPT, os pacientes eram sintomáticos (apesar da rigorosa terapia clínica otimizada), tinham IM mais importante, VE menores e melhor função sistólica em relação ao MITRA-FR. No estudo MITRA-FR, os pacientes apresentavam IM menos importante, VE maiores e com pior função sistólica, em estágio mais avançado de cardiomiopatia. A disfunção ventricular foi o principal causador da IC e de desfechos clínicos e, por isso, a terapia com MitraClip® pode não ser tão benéfica.^30^

A identificação antecipada da IM secundária antes que o VE se dilate em excesso é crucial. Embora seja considerada sucesso de procedimento IM residual ≤ +2 (moderada), a meta do procedimento deve ser IM ≤ +1 (leve) e a implantação de clipes adicionais deve ser considerada com o objetivo de atingir essa meta. Tendo em vista os achados do COAPT e MITRA-FR, acreditamos que os dois estudos são complementares. Esperamos que o estudo randomizado RESHAPE-HF (A Randomized Study of the MitraClip Device in Heart Failure Patients with Clinically Significant Functional Mitral Regurgitation),^31^ ainda em recrutamento e com o mesmo critério de inclusão do COAPT, possa fornecer um entendimento ainda maior na fisiopatologia da IM secundária, especialmente após dados conflitantes.

**Figura 1:**
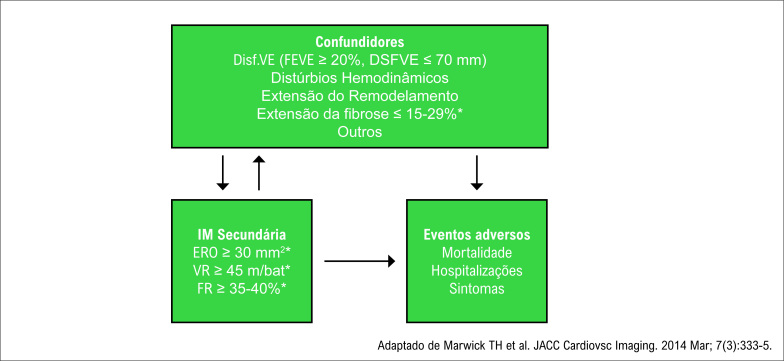
A complexidade da insuficiência mitral funcional e a seleção do candidato ideal* para o implante de MitraClip®. Disf.: disfunção; DSFVE: diâmetro sistólico final do ventrículo esquerdo; ERO: orifício regurgitante efetivo; FEVE: fração de ejeção do ventrículo esquerdo; FR: fração regurgitante; VR: volume regurgitante.

Ainda estamos no processo de definição do candidato ideal para o tratamento da insuficiência mitral secundária por MitraClip®. É possível que o tamanho do infarto e/ou fibrose também possam ajudar na melhor seleção desses pacientes.^31^^,^^32^ Além disso, é necessário certificar que a gravidade da IM é puramente atribuível à gravidade da IM e não a outros fatores de risco e fatores confundidores. O estudo COAPT reforça o importante papel da IM na fisiopatologia da IC sistólica e, com a seleção apropriada de pacientes – excluindo os VE maiores, mais doentes, com maior área de fibrose e IM moderada e selecionando pacientes nos quais a IM seja tão severa que contribua para a gravidade da doença –, o tratamento percutâneo da IM secundária por MitraClip® pode ser benéfico, desde que obedeça aos seguintes critérios (Figura 1):

1. Garantia de que a gravidade da IM seja puramente atribuível à gravidade da IM e não a outros fatores que influenciam a IM (idade, comorbidades, outras doenças cardíacas, grau de disfunção ventricular, extensão da fibrose, extensão do remodelamento).
2. Avaliação da gravidade da IM pela integração de múltiplos parâmetros além do ERO: VR, fração regurgitante e possível quantificação da extensão da área de fibrose.
3. IM ≥ +3 (moderada a importante), definida como ERO ≥ 30 mm^2^ e/ou VR de 45 mL por batimento.
4. FEVE de 20% a 50% e diâmetro sistólico do VE <70 mm.
5. Sintomas de IC, apesar da terapia clínica otimizada (máxima dose tolerada), incluindo terapia de ressincronização e revascularização coronariana, se apropriada.
6. Grupo intervencionista com experiência, com sucesso técnico na redução da IM ≥ +2 maior que 95%.
7. Presença de um time multidisciplinar (*heart team*) para manejo, tratamento e otimização da IC.
8. Após intervenção, acompanhamento de perto das medicações e do *status* volêmico.
9. Identificação antecipada da IM secundária e encaminhamento a um time multidisciplinar (*heart team*) antes que o ventrículo dilate muito ou o paciente seja hospitalizado, necessitando de cuidados intensivos ou de suporte inotrópico.
