# Supplementary material for: Tratamento Percutâneo da Insuficiência Mitral Secundária por MitraClip: Mitra-FR versus COAPT
Source: Arq Bras Cardiol. 2021 May 6;116(5):1011–8. [Article in Portuguese] doi: 10.36660/abc.20200063 (PMC8121475; doi:10.36660/abc.20200063)
Supplement: Translation

# Translation: Percutaneous Treatment of Secondary Mitral Regurgitation by MitraClip: Mitra-FR vs. COAPT

**Authors:** Sergio Barros-Gomes, Flávio Tarasoutchi, Ana Clara Tude Rodrigues, Lara Ferreira Nhola, Pedro Alves Lemos, Samira Saady Morhy, Claudio Henrique Fischer, Marcelo Luiz Campos Vieira

**Affiliations:** 2 São PauloSP Brazil Hospital Israelita Albert Einstein, São Paulo, SP - Brazil; 1 São PauloSP Brazil Instituto do Coração da Universidade de São Paulo, São Paulo, SP - Brazil; 3 São PauloSP Brazil Universidade Federal de São Paulo, São Paulo, SP - Brazil

**Keywords:** Heart Failure, Mitral Valve, Insufficiency, Echocardiography/methods, Clinical Trials

## Introduction

Secondary or functional mitral regurgitation (MR) is due to changes in the geometry of the left ventricle (LV) secondary to ventricular dysfunction.^1^ It occurs when an ischemic heart disease or a dilated cardiomyopathy of any etiology causes dilation of the LV, dilation of the mitral ring, and/or displacement of the papillary muscle, resulting in poor coaptation of the valve cusps and valve regurgitation.^2^ the American Heart Association indicates that 16,250 per million North Americans have secondary MR,^3^^,^^4^ totaling more than 5 million cases in the United States of America alone, and this number is estimated to be even greater due to the continued growth and aging of the population. This is noteworthy, as secondary MR causes a poor prognosis and is an independent predictor of mortality.^5^^,^^6^

For many years, the mechanical intervention of secondary MR (surgical or percutaneous) has been restricted to cases refractory to conventional clinical treatment,^7^^,^^8^ with evidence mainly supported by two important studies conducted by the Cardiothoracic Surgical Trials Network group.^9^^,^^10^ The first study^9^ randomized 301 patients with moderate ischemic MR and found no differences in ventricular geometry between patients who undergone surgical myocardial revascularization versus the combination of surgical revascularization and mitral valve repair. The second study^10^ surveyed 251 patients with severe MR and found no differences regarding mortality, in addition to the greater recurrence of mitral regurgitation and complication rates among patients treated with mitral valve repair versus valve replacement. Considering these two studies, the recommendations of the American Heart Association/American College of Cardiology^7^ and the Brazilian Guidelines for Valvular Heart Disease^8^ classified surgical or percutaneous mitral valve intervention as a Class IIb indication.

Until recently, no randomized trial had compared percutaneous secondary MR intervention with the conventional clinical treatment. In 2018, the conduct towards secondary MR decisively changed with the presentation of two randomized clinical trials: the *Multicentre Study of Percutaneous Mitral Valve Repair MitraClip Device in Patients With Severe Secondary Mitral Regurgitation* (MITRA-FR)^11^ and the *Cardiovascular Outcomes Assessment of the MitraClip Percutaneous Therapy for Heart Failure Patients with Functional Mitral Regurgitation* (COAPT).^12^ These studies evaluated the efficacy and safety of two therapeutic strategies in patients with severe secondary MR – percutaneous therapy with MitraClip® together with optimized clinical treatment versus optimized clinical treatment alone.

In this article, the main similarities and differences between both studies will be addressed, in addition to considering the application of this procedure to the clinical practice, including the ideal profile of the candidate for the procedure. Table 1

**Table 1:** Characteristics of recruitment, randomization and clinical follow-up

Variable	MITRA-FR	COAPT
Patients, n	304	614
Patients Intervention/Control, n	152/152	302/312
Study period, years	2013-2017	2012-2017
**Inclusion criteria**		
ERO, mm^2^	> 20	> 30
RV, mL/beat	30	45
LVEF, %	15-40	20-50
LVESD, mm	NA	≥ 70
Daily medications	Adjusted in each group according to clinical practice	Maximum stabilized dose and resynchronization therapy if appropriate
Symptoms	NYHA II, III, IV	NYHA II, III, IV

LVESD: left ventricular end-systolic diameter; ERO: effective regurgitant orifice area; LVEF: left ventricular ejection fraction; NYHA: New York Heart Association; RV: regurgitant volume.

### MITRA-FR

MITRA-FR was a multicenter study conducted in 37 French centers that randomized 304 patients with severe secondary MR, symptomatic systolic heart failure (HF), and left ventricular ejection fraction (LVEF) between 15% and 40% in two therapeutic strategies, in a 1:1 ratio, allocated for percutaneous treatment with MitraClip® together with optimized clinical treatment (intervention group; 152 patients) versus isolated optimized clinical treatment (control group; 152 patients).^11^ Severe secondary MR was defined as having an effective regurgitant orifice area (ERO) > 20 mm^2^ or regurgitant volume (RV) > 30 mL per beat. The primary endpoint was mortality from any cause or hospitalization for HF within 12 months. Patients in both groups showed an improvement in the functional class, but with no significant difference between the two groups. Finally, there was no significant difference in the composite primary endpoint (54.6% vs. 51.3%, respectively; p = 0.53), mortality rate (24.3% vs. 22.4%; p > 0.05), and hospitalization rate (48.7% vs. 47.4%; p > 0.05) between the intervention versus control group during 1 year of follow-up. Likewise, there was no significant difference in the composite primary endpoint (63.8% vs. 67.1%, respectively; p > 0.05), mortality rate (34.9% vs. 34.2%; p > 0.05), and hospitalization rate (55.9% vs. 61.8%; p > 0.05) between the intervention versus control group during 2 years of follow-up.^13^ The authors concluded that MitraClip® is safe and effective in secondary MR compared with optimized clinical treatment, but with no improvement in survival or reduced hospitalization for HF in patients with secondary MR and systolic HF.

### COAPT

COAPT was a multicenter study that randomized 614 patients in 78 North American and Canadian centers with symptomatic systolic HF and moderate to severe (3+) or severe (4+) secondary MR, defined as ERO > 30 mm^2^ or RV > 45 mL per beat, with LVEF ≥of 20% (mean LVEF of 31.3 ± 9.3%), in the ratio of 1: 1, allocated for percutaneous treatment with MitraClip® together with optimized clinical treatment (intervention group; 302 patients) versus isolated optimized clinical treatment (control group; 312 patients).^12^ Symptomatic HF was defined as symptoms of HF despite the maximum tolerated drug dose. The primary efficacy endpoint was hospitalization for HF within 24 months and the primary safety endpoint was an event free of complications related to MitraClip® at 12 months. The annual rate of hospitalization for HF within 24 months was 35.8% per patient/year in the intervention group versus 67.9% in the control group (*hazard ratio* 0.53; 95%CI 0.40-0.70; p <0.001). The percentage of event-free complications related to the device in 12 months was 96.6% (p < 0.001), whereas death from any cause in 24 months occurred in 29.1% in the intervention group compared with 46.1% in the control group (*hazard ratio* 0.62; 95%CI, 0.46-0.82; p < 0.001). The intervention group not only reduced the rate of hospitalizations for HF by 47%, but also reduced mortality by 38%. Reduction in the absolute risk of all-cause mortality in the MitraClip® group was 17%, and the number necessary for treatment in order to prevent death in 2 years was 5.9; to prevent hospitalization for HF in 2 years, it was 3.1. The authors concluded that the MitraClip® combined therapy and optimized clinical treatment for patients with symptomatic systolic HF and moderate to severe or severe MR reduces the number of hospitalizations for HF and all-cause mortality in 2 years when compared with exclusively optimized clinical treatment. Tables 2 and 3 compare the characteristics and clinical outcomes between both studies.

**Table 2:** Clinical and echocardiographic characteristics

Variable^a^		MITRA-FR	COAPT
Clinical			
Age, years			
	– MitraClip group	70 ± 10	72 ± 12
	– Control group	71 ± 10	73 ± 10
Sex, male, n (%)		120 (79)	201 (67)
		107 (70)	(62)
NYHA, %			
	-I	0	0.2
	-II	32.9	39
	-III	58.5	52.5
	-IV	8.6	8.3
Ischemic cardiomyopathy			
	– MitraClip group	95 (62.5)	184 (60.9)
	– Control group	85 (56.3)	189 (60.6)
Previous myocardial revascularization			
	– MitraClip group	71 (46.7)	PCI: 130 (43.0) CABG: 121 (40.1)
	– Control group	62 (42.4)	PCI: 153 (49.0) CABG: 126 (40.4)
Previous cardiac resynchronization			
	– MitraClip group	46 (30.5)	115 (38.1)
	– Control group	35 (23.0)	109 (34.9)
Surgical Risk			
	- STS score	NA	8.2 ± 5.9%
	- EuroScore II	6.2 (3.5-11)	NA
**Echocardiography**			
MR severity, %			
	- ERO 20-29 mm^2^ (moderate)	157 (52.2)	80 (13.5)
	- ERO 30-39 mm^2^ (moderate/severe)	95 (31.6)	270 (45.7)
	- ERO ≥ 40 mm^2^ (severe)	49 (16.3)	241 (40.8)
ERO, mm^2^		31 ± 10	41 ± 15
LVEDVI, mL/m^2^		135 ± 35	101 ± 34
LVEF, %		33 ± 7	31 ± 9

ERO: effective regurgitant orifice area; LVEF: left ventricular ejection fraction; NA: not applicable; NYHA: New York Heart Association; STS: Society of Thoracic Surgeons – risk of death within 30 days after mitral valve replacement; LVEDVI: left ventricular end-diastolic volume index.

a Categorical variables are reported in numbers (percentages); continuous variables are reported as mean ± standard deviation [SD] and median (interquartile range).

**Tabela 3:** Desfecho clínico

Variable^a^		MITRA-FR	COAPT
MitraClip Group only, n			
	- Complications in the procedure	21 (14.6)	25 (8.5)
	- MR ≥ +2 on discharge	93 (24.4)	214 (17.7)
	- MR ≥ +2 in 2 years	48 (49.5)^b^	26 (22.8)
Mortality from any cause in 2 years, n			
	– MitraClip group	53 (34.9)	80 (29.1)
	– Control group	52 (34.2)	121 (46.1)
	p value	>0.05	<0.001
Hospitalizations for CHF in 2 years, n			
	– MitraClip group	85 (55.9)	92 (35.7)
	– Control group	94 (61.8)	151 (56.7)
	p value	>0.05	<0.001
Mortality from any cause or hospitalizations associated with HF in 2 years, n			
	– MitraClip group	97 (63.8)	129 (45.7)
	– Control group	102 (67.1)	191 (67.9)
	p value	>0.05	<0.001

HF: heart failure; CHF: congestive heart failure; MR: mitral regurgitation.

a Categorical variables are reported in numbers (percentages).

b MR +2 in 1 year.

### Main Similarities and Differences

Both trials had conflicting results, with COAPT showing benefit from MitraClip® versus drug therapy, whereas MITRA-FR showed no benefit related to MitraClip®. Undoubtedly, these two studies have changed the researchers' understanding of secondary MR. But why did they show significantly different results? Why did the COAPT study have a positive result, whereas the MITRA-FR was neutral? The answer to this question is probably multifactorial and includes differences in patients' selection, optimization of drug therapy, grade of MR, and ventricular remodeling.

#### Recruitment:

The COAPT recruitment was more selective compared with MITRA-FR, considering that its recruitment was slower and more prolonged. The number of patients was different in both studies: COAPT recruited about 300 patients in each group, and MITRA-FR, around 150 patients. Perhaps the sample size of the MITRA-FR population, after excluding patients with incomplete follow-up, may not have been sufficient to detect statistical significance and thus avoid type II error, especially in relation to secondary endpoints. In the COAPT study, the number of hospitalizations between the two therapeutic strategies has diverged since the beginning of the follow-up, partially explained by the more rigorous drug treatment.

#### Grade of MR:

In MITRA-FR, the mean ERO was 31 mm^2^, whereas COAPT had a mean ERO of 41 mm.^2^ Although the inclusion criterion for both studies was MR with grade from moderate to severe, the COAPT study followed the North American recommendations of 2008,^14^ which classifies moderate to severe MR when the ERO is ≥ 30 mm^2^ and/or 45 mL RV; MITRA-FR followed the European recommendations of 2012:^15^ ERO ≥ of 20 mm^2^ and/or 30 mL RV classified as moderate to severe MR. This disagreement is based on the concept that mortality in patients with secondary MR is significantly higher for lower levels of ERO and RV.^16^^,^^17^ However, the mechanism of functional MR is complex and it is unknown whether moderate ERO or RV actively work as causes of ventricular remodeling and dysfunction, or if they are mere markers resulting from incipient cardiomyopathy. Subsequent guidelines returned ERO and RV to their usual values; based on current recommendations, ERO of 30 mm^2^ is considered moderate, whereas ERO of ≥ 40 mm^2^ is considered severe.^8^^,^^18^ New studies suggest that the unified approach, based on the integration of ERO, RV, and regurgitant fraction (RF), may be an excellent discriminator of severe secondary MR when compared with the algorithms established in the latest guidelines and, therefore, an excellent identifier of patients at high risk.^19^ Hence, a significant number of patients (52%) with moderate MR (ERO of 20-30 mm^2^) were recruited for MITRA-FR, whereas only 14% of patients with these characteristics were recruited for COAPT. Regarding severe MR, (ERO ≥ of 40 mm^2^), only 16% of MITRA-FR patients had severe MR versus 41% of COAPT. The findings of both studies suggest that the benefit of MitraClip® is greater for patients with ERO > 40 mm^2^ (i.e., truly severe MR).

#### Ventricular Remodeling:

The mean left ventricular end-diastolic volume index (LVEDVI) of the patients in the MITRA-FR study was 135 mL/m^2^ compared with 101 mL/m^2^ of the COAPT. The LV was significantly greater in MITRA-FR, characterizing more remodeled ventricles, in more advanced stages of cardiomyopathy. This difference is probably due to the exclusion of patients with severe dilation/dysfunction in COAPT (LV systolic diameter > 70 mm), whereas in MITRA-FR there was no such limitation. The inclusion criterion for LVEF between the two studies was also different: COAPT included patients with LVEF of 20-50% versus LVEF of 10-40% in MITRA-FR. Interestingly, a subgroup of patients in the COAPT study who did not benefit from treatment with MitraClip® (number of hospitalizations associated with HF within 12 months) consisted of patients with ERO and LVEDVI relatively similar to those recruited in the MITRA-FR study (ERO ≥ of 30 mm^2^ and LVEDVI > 96 mL/m^2^).^20^ These facts suggest that patients with moderate MR, markedly more dilated LV, and with greater dysfunction may not be ideal candidates for the treatment with MitraClip®. In fact, the high recurrence of MR and the worst clinical outcome had been previously reported in the surgical correction of patients with ischemic MR, ventricular dilation (LV diastolic diameter > 65 mm), and severe LV dysfunction (LVEF < 20% and LV systolic diameter > 55 mm).^21^^,^^22^ In the MITRA-FR study, cardiomyopathy was possibly the main cause of HF symptoms and, consequently, the determinant of the unfavorable clinical outcome, i.e., MR was merely a factor secondary to ventricular remodeling. On the other hand, in the COAPT study, HF was partly due to MR and, therefore, the grade of MR in the COAPT trial was higher, while cardiomyopathy was less advanced (smaller LV and greater LVEF).

#### Drug Therapy and Therapeutic Optimization:

In the COAPT study, the patient inclusion criterion was symptomatic systolic HF despite the maximum tolerated drug dose, use of cardiac resynchronization therapy, use of defibrillators, and myocardial revascularization therapy (if appropriate). Patients were clinically optimized prior to recruitment and only a few medication adjustments were made during follow-up. Conversely, in the MITRA-FR study, it was not possible to optimize the medication in all patients before randomization and multiple readjustments during follow-up. In MITRA-FR, the medication was adjusted by the researchers, whereas in COAPT the medication was more rigorously adjusted by a group of specialists who supervised the maximum tolerated dose, before and after the intervention. The initial dosage and adjusted doses of each medication were accounted for in the COAPT study. Certainly, this rigor in terms of dosage and drug optimization implemented in the COAPT study does not reflect the daily clinical practice.

#### Success in Reducing MR:

At the end of 12 months, 83% of MITRA-FR patients had MR ≤ +2 (moderate) compared with 95% of COAPT patients. Consequently, 17% of MITRA-FR patients had MR ≥ +3 (moderate/severe) in 12 months compared with 5% of COAPT patients. The COAPT study had a more aggressive strategy in terms of implanted clips when compared with MITRA-FR (use of one clip in 36% of cases for COAPT vs. 46% for MITRA-FR; two clips in 55% of COAPT cases vs. 46% for MITRA-FR; three clips in 55% of COAPT cases vs. 9% of MITRA-FR; four clips in 0.3% of COAPT patients vs. 0% for MITRA-FR). The higher success rate in reducing MR may be associated with favorable results.

#### Pathophysiology:

Divergences in terms of pathophysiology have been elegantly demonstrated by Packer and Grayburn et al.,^23^ who presented the concept of proportionate MR versus disproportionate MR based on the combination of ERO and end-diastolic volume (EDV) – ERO/EDV ratio. Assuming a LVEF of 30% and a regurgitant fraction of 50% (profile of patients in the trials), the authors graphically showed that an ERO of 30 mm^2^ and a higher EDV (between 220-240 mL) could result in a regurgitant fraction of 50%, and a 20 mm^2^ ERO and normal EDV could result in a 50% regurgitant fraction.^23^ The authors suggest that percutaneous treatment of the mitral valve by MitraClip® is more beneficial for patients with disproportionate MR as for the size of the LV, i.e., when the MR is greater than expected for an dilated LV, treatment with MitraClip® may have a more favorable result (larger ERO and lower LV). In contrast, proportionate MR would represent sicker patients, with larger ventricles and a lower grade of MR. In other words, patients with late-stage cardiomyopathy selected for interventional treatment.

Nevertheless, Gaasch and Meyer et al.^24^ suggested that the severity of MR between the two trials is actually quite similar. The authors argue that the pathophysiology of MR is better described by RV (or the regurgitant fraction) than by ERO. RV is determined by ERO and the magnitude and duration of the systolic pressure gradient through the regurgitant valve, i.e., ERO is only one of the determining variables of RV. In fact, RV affects the LV size at a given LVEF, and has a direct relationship with the EDV. Thus, they graphically proposed that the association between severity of MR and LV size should be based on the ratio between RV and EDV – RV/EDV ratio –, with its quotient being uniformly corrected, making it a dimensionless index. Assuming a 50% regurgitant fraction in the COAPT study (assumption based on LVEF and echocardiographic data) and a 53% regurgitant fraction provided in MITRA-FR, the RV/EDV ratio was 0.18 and 0.15, respectively. These coefficients of proportionality are relatively low (both < 0.20) and similar to the values reported in previous studies on secondary MR, reflecting a proportionally small contribution of RV to a large EDV. Thus, there is a disproportionate increase in the LV in the patients' profile of the two trials typically observed in patients with secondary MR (disproportionate MR) compared with patients with primary MR (EDV proportional to RV).

#### Underestimated volumes:

In the COAPT study, patients had a mean ERO of 41 ± 15 mm^2^, which corresponds to a RV of at least 45-60 mL. The total stroke volume of the LV in the COAPT study was 57 mL (LV end-diastolic volume subtracted from the end-systolic volume), which is totally incompatible to maintain a satisfactory cardiac output. Assuming a 57-mL total stroke volume of the LV, RV is hence the total stroke volume of the LV subtracted from the stroke volume in the outlet (i.e., the total stroke volume of the LV is equal to the mitral RV plus the stroke volume in the left ventricular outflow tract, the forward stroke volume ranges from 0 to 15 mL, which would be incompatible with life. It is clear that EDV in the COAPT study is underestimated. If we assume a 41-mm^2^ ERO and a 60-mL RV (similar to the COAPT study), EDV should be greater than 300 mL (assuming a 50% regurgitant fraction and 31% LVEF as reported in the study). Nevertheless, the LV diastolic diameter was smaller in the COAPT study (mean of 69 mm in MITRA-FR versus 62 mm in COAPT), confirming smaller LVs.

In fact, the quantification of secondary MR using two-dimensional echocardiography is challenging due to the numerous limitations of the method itself, in addition to the complex pathophysiology of MR. In patients with functional MR, ERO and RV, according to the PISA method, are mostly underestimated with values of cardiac resonance^25^ and three-dimensional echocardiography.^26^ The non-circular orifice and the dynamic behavior of MR significantly contribute to these differences. Perhaps the regurgitant fraction can overcome these limitations and corroborate as an essential variable of severity, in addition to its important prognostic role.^12^ The regurgitant fraction is calculated by the ratio between RV and total stroke volume (RV/total stroke volume) – which, despite being variables dependent on LV loading conditions, size, and function, its quotient is uniformly corrected by these parameters, thus being a more robust indicator.^27^

#### *Other factors*:

It is worth noting that unlike primary MR, in which severity is purely quantified based on the MR grade, secondary MR is complex, heterogeneous, and influenced by several factors: age, underlying disease, comorbidities, LV remodeling, extent of infarction, hemodynamic disorders, among others.^28^ In the COAPT study, the combined outcome mortality or hospitalization for heart failure in the group that was treated with MitraClip® was relatively significant (46%). This shows that, regardless of valve repair, these patients continue to have a poor prognosis, considering that most part of the risk is related to these factors.

Likewise, Cavalvante et al.^29^ demonstrated that the regurgitant fraction and the infarction size measured in patients with ischemic heart disease consist in important risk stratifications that go beyond the size of the LV and other clinical variables. The authors also reported that the prognosis of these patients is worse as the infarction size and the grade of MR increase. Noteworthily, the extent of fibrosis was not measured in MITRA-FR and COAPT studies, but the authors of the present article believe that it certainly had a clinical impact on the outcome of these studies. Perhaps patients with larger hearts and a larger area of infarction cannot benefit from MitraClip®. Likewise, it is possible to speculate that patients in the MITRA-FR study had a larger area of fibrosis and, therefore, less benefit from the MitraClip® therapy. New studies correlating clinical outcomes in patients treated with MitraClip® and the extent of fibrosis would be interesting.

### Implications for Clinical Practice

Both studies evaluated the same clinical entity: functional or secondary MR. In the COAPT study, patients were symptomatic despite rigorous optimized clinical therapy, had more severe MR, smaller LV, and better systolic function compared with MITRA-FR. In the MITRA-FR study, patients had less severe MR, larger LV, and worse systolic function, in a more advanced stage of cardiomyopathy. Ventricular dysfunction was the main cause of HF and clinical outcomes and, therefore, therapy with MitraClip® may not be considerably beneficial.^30^

The early identification of secondary MR before LV is over-dilated is crucial. Although being considered a successful procedure a residual MR ≥ +2 (moderate), the goal of the procedure should be MR ≤ +1 (mild), and the implantation of additional clips should be taken into account in order to achieve this goal. Considering the findings of COAPT and MITRA-FR studies, the authors of the present article believe that both studies are complementary. It is expected for the randomized study RESHAPE-HF (*A Randomized Study of the MitraClip Device in Heart Failure Patients With Clinically Significant Functional Mitral Regurgitation*),^31^ still in the recruitment step and with the same inclusion criteria as COAPT, to provide an even greater understanding of the pathophysiology of secondary MR, especially after conflicting data.

Moreover, the authors are currently in the process of defining the ideal candidate for the treatment of secondary mitral regurgitation by MitraClip®. The size of infarction and/or fibrosis may also assist in better selecting these patients.^31^^,^^32^ In addition, the severity of MR must be confirmed as being purely due to the severity of MR and not to other risk and confounding factors. The COAPT study emphasizes the important role of MR in the pathophysiology of systolic HF and, with appropriate patient selection, excluding those with larger LV, sicker, with larger area of fibrosis, and moderate MR, and selecting patients with very severe MR in such a way it contributes to the severity of the disease itself, percutaneous treatment of secondary MR by MitraClip® can be beneficial as long as it meets the following criteria (Figure 1):

**Figure 1:** The complexity of functional mitral regurgitation and the selection of the ideal candidate* for the MitraClip® implant. Dysf.: dysfunction; LVESD: left ventricular end-systolic diameter; ERO: effective regurgitant orifice area; LVEF: left ventricular ejection fraction; RF: regurgitant fraction; RV: regurgitant volume.

To ensure that the severity of MR is purely attributable to the severity of MR and not to other factors that influence MR (age, comorbidities, other heart diseases, degree of ventricular dysfunction, extent of fibrosis, extent of remodeling).

Assessment of the severity of MR by integrating multiple parameters in addition to ERO: RV, regurgitant fraction, and possible quantification of the extent of the fibrosis area.

MR ≥ +3 (moderate to severe), defined as ERO ≥ 30 mm^2^ and/or 45 mL RV per beat.

LVEF of 20-50% and LV systolic diameter < 70 mm.

Symptoms of HF despite optimized clinical therapy (maximum tolerated dose), including cardiac resynchronization therapy and myocardial revascularization, if appropriate.

Experienced interventionist group, with technical success in reducing MR ≥ +2 greater than 95%.

The presence of a multidisciplinary team (heart team) for the management, treatment, and optimization of HF.

After intervention, close monitoring of medications and volume status.

Early identification of secondary MR and referral to a multidisciplinary team (heart team) before over-dilation of the ventricle or the patient is hospitalized, requiring intensive care or inotropic support.
